# Transcriptome analysis of malate-induced *Schizochytrium* sp. FJU-512 reveals a novel pathway for biosynthesis of docosahexaenoic acid with enhanced expression of genes responsible for acetyl-CoA and NADPH accumulation

**DOI:** 10.3389/fmicb.2022.1006138

**Published:** 2022-10-10

**Authors:** Mingliang Zhang, YangLe Gao, Cui Yu, Jun Wang, Kexin Weng, Qin Li, Yongjin He, Zheng Guo, Huaidong Zhang, Jianzhong Huang, Li Li

**Affiliations:** ^1^Engineering Research Center of Industrial Microbiology of Ministry of Education, Fujian Normal University, Fuzhou, China; ^2^College of Life Sciences, Fujian Normal University, Fuzhou, China; ^3^Department of Biological and Chemical Engineering, Aarhus University, Aarhus, Denmark

**Keywords:** docosahexaenoic acid, comparative transcriptomics, malate, fatty acid metabolism, acetyl-CoA

## Abstract

*Schizochytrium* is one of the few oleaginous microalgae that produce docosahexaenoic acid (DHA)-rich lipids. In this study, global changes in gene expression levels of *Schizochytrium* sp. FJU-512 cultured with malate in a 15 l-bioreactor was analyzed using comparative transcriptomics. The changes were found mainly in the genes involved in oxidative phosphorylation, β-oxidation, and pentose phosphate pathways. Consequently, the global changes in genes associated with the pathways could lead to an increase in the influx throughputs of pyruvate, branched-chain amino acids, fatty acids, and vitamin B6. Our transcriptome analysis indicated pyruvate dehydrogenase E2 component and acetolactate synthase I/II/III large subunit as major contributors to acetyl-CoA biosynthesis, whereas glucose-6-phosphate dehydrogenase was indicated as the major contributor to the biosynthesis of NADPH. An increase in DHA titer of up to 22% was achieved with the addition of malate to the fed-batch culture of *Schizochytrium* sp. FJU-512. This study provides an alternate method to enhance DHA production in *Schizochytrium* sp. FJU-512 through malate induced upregulation of genes responsible for acetyl-CoA and NADPH biosynthesis.

## Introduction

Docosahexaenoic acid (DHA, C22: 6n-3), an omega-3 polyunsaturated fatty acid (PUFA), plays an important role in alleviating ailments such as cardiovascular diseases, cancer, inflammation, hypertension, Alzheimer’s disease, and peroxisomal disorders (Li et al., 2021). DHA is supplemented by dietary intake because biosynthesis from precursors in mammals is limited; hence, there is a high commercial demand for DHA-rich oil (Tan et al., 2020). Currently, nutraceutical industries use cold-water fish as the main source for DHA production (Pike and Jackson, 2010). However, extraction of DHA from cold water fishes is disadvantageous owing to inconsistent product quality, undesirable smell, and the presence of environmental pollutants—all of which contradict the principle of healthy eating. Therefore, there is an urgent need to explore alternative sources of DHA that offer a sustainable, safe, and economical mode of DHA production. Several microbial species/strains that synthesize large amounts of DHA possess a high DHA to total fatty acid ratio, which makes them ideal candidates for the production of high-quality DHA, and *Schizochytrium* is one of the excellent DHA producers (Oliver et al., 2020; Chi et al., 2022). Owing to the high amounts obtained from the *Schizochytrium* cultures, DHA could be used as a low-cost nutraceutical for the food and feed markets.

Both bioprocess improvement, as well as cost reduction, are crucial parameters for large-scale production of DHA from microbial sources. Prior to optimization of such parameters, the microbial biosynthetic pathways of DHA must be thoroughly understood. DHA biosynthesis occurs mainly through two pathways: one is the aerobic pathway involving fatty acid desaturase/elongase, in which fatty acids are alternately catalyzed by desaturase and elongase to synthesize DHA (Uttaro, 2006); the other is the anaerobic polyunsaturated fatty acid synthase (PUFA-S) pathway, which requires fewer reducing equivalents of nicotinamide adenine dinucleotide phosphate (NADPH) to produce specific fatty acid components comprising only of DHA (Metz et al., 2001). The ability of microorganisms to accumulate large quantities of fatty acids depends on two prerequisites: a continuous supply of the precursor acetyl-CoA in the cytoplasm, and a sufficient supply of the reductant NADPH (Ratledge, 2014). Malate is an important molecule involved in the tricarboxylic acid (TCA) and transhydrogenase cycle in the fatty acid biosynthesis (Mizuarai et al., 2005). The addition of malate (4 g/l) increased the DHA content of total fatty acids from 35 to 60% during the rapid lipid accumulation stage in *Schizochytrium* sp. HX-308 (Ren et al., 2009). To date, the mechanism of DHA accumulation induced by malate in *Schizochytrium* sp. has not been systematically analyzed; the role of malate in fatty acid biosynthesis and lipid accumulation is still unclear.

With the rapid development of next-generation sequencing (NGS) technologies such as transcriptome sequencing, it is possible to obtain gene expression profiles of lipid producers under different environmental stress condition (Blow, 2009). *Schizochytrium* sp. S056 uses glycerol to produce docosahexaenoic acid (Chen et al., 2016), lipid synthesis in *Aurantiochytrium* sp. occurs under limited nitrogen supply (Heggeset et al., 2019), and DHA accumulations in *Schizochytrium* sp. accompany with phenotypic heterogeneity (Bao et al., 2022). However, little is known about transcriptome variations in response to the addition of different levels of malate for DHA synthesis in *Schizochytrium* sp. The objective of this study is to elucidate the molecular mechanisms associated with DHA biosynthesis using comparative transcriptome analysis of *Schizochytrium* sp. FJU-512 with the addition of malate.

In this study, RNA-seq-based transcriptomic analysis of *Schizochytrium* sp. FJU-512 in fed-batch culture was used to understand the molecular mechanism underlying the improvement in DHA accumulation by malate. Global gene expression analysis indicated that increased malate supply led to increased expression of genes related to NADPH and acetyl-CoA metabolism, the citrate cycle (TCA cycle), oxidative phosphorylation, nitrogen metabolism, and vitamin B6 metabolism. Several genes were involved in pathways that were different from those reported to play roles in an enhanced supply of acetyl-CoA and NADPH and other cellular processes to promote the accumulation of DHA by transcriptome analysis in *Schizochytrium* sp. FJU-512, when malate was added. These results offer fresh data on several genes and enzymes for metabolic engineering lipid synthesis in oleaginous microalgae.

## Materials and methods

### Microorganisms and culture conditions

*Schizochytrium* sp. FJU-512, was stored in our laboratory with 25% (v/v) glycerol at-80°C until further use. Culture media and conditions were maintained as described previously (Qi et al., 2017). 1 g/l, 3 g/l, and 5 g/l of malate were added at 36 h, 60 h, and 84 h of flask shaking, respectively. The strain was grown for three generations and then seeded in 8 l of fermentation medium in a 15 l bioreactor (Zhenjiang East Biotech Equipment and Technology Co., Ltd., China). A one-time 3 g/l malate supply was given to 15 l of fed-batch fermentation at 60 h. Fed-batch cultures were grown at 28°C with an initial aeration rate of 1 vvm; the pH was maintained at 5.7 ± 0.1 using ammonia. To maintain a residual glucose concentration of 20–30 g/l, 80% glucose solution was added continuously to the bioreactor. Fermentation was performed for 120 h.

### Cell dry weight, total lipids, and fatty acid analyses

Cell dry weight was determined gravimetrically by subjecting a 10 ml culture to centrifugation (Eppendorf 5,810 R, Eppendorf, Germany) for 5 min at 8,000 × *g*. The pelleted cells were washed with saline and then transferred to a new microcentrifuge tube and dried at 85°C for 48 h to obtain the dry weight. Lipid extraction and fatty acid methyl ester (FAME) preparation were performed as reported previously (Qi et al., 2017). FAME samples were analyzed using GC–MS (Agilent 6,890 N/5975C, Agilent, United States) equipped with an HP-INNOWAX capillary column (30 m × 0.25 mm × 0.25 μm). The oven temperature was increased from 150°C to 220°C at a rate of 10°C/min and thereafter at a rate of 2°C/min until it reached 230°C, which was maintained for 5 min. The concentration of each fatty acid was calculated based on the total peak area compared to that of the internal standard (0.5 mg/l nonadecanoic acids; Zeng et al., 2021).

### RNA isolation and library preparation

Total RNA was extracted using TRIzol-A+ reagent (TIANGEN BIOTECH, Beijing, China) and then treated with RNase-free DNase I (TaKaRa, Dalian, China), according to the manufacturer’s protocol. RNA concentration was measured using a Nanodrop Qubit 2.0 Fluorometer (Life Technologies, CA, United States). RNA quality was evaluated using Agilent Bioanalyzer Model 2,100 (Agilent Technologies, Palo Alto, Canada). According to the Illumina transcriptome sequencing protocol, samples with an RNA integrity number (RIN) value >7.5 were deemed acceptable. All sequencing reads were submitted to the Sequence Read Archive (SRA) and National Center for Biotechnology Information (NCBI).

### Transcriptome sequencing and assembly

The mRNA was prepared at 72 h and 86 h, captured using oligo dT magnetic beads, and fragmented using fragmentation buffer. The fragmented mRNA was used as a template for cDNA synthesis. First-strand cDNA was synthesized using reverse transcriptase and random primers, followed by second-strand cDNA synthesis using DNA polymerase I and RNase H. Samples were purified using the QIA quick PCR kit and eluted with elution buffer for end repair and sequencing adapter joining. The cDNA fragments were separated by agarose gel electrophoresis, and fragments of 100 ± 300 bp were amplified by PCR to create cDNA libraries. The constructed cDNA libraries were sequenced using an Illumina HiSeq 4,000 platform. The fastq-format raw reads were first processed using in-house Perl scripts. In this step, clean reads were obtained by removing reads containing adapters, poly-N, and low-quality reads from raw reads. At the same time, the Q30 and GC contents of the clean data were calculated. Clean reads were assembled using Trinity (Haas et al., 2013).

### Functional annotation and differentially expressed gene identification

The unigene function was annotated based on the following databases: Nr (NCBI non-redundant protein sequences), Nt (NCBI non-redundant nucleotide sequences), Pfam (Protein family), KOG/COG (Clusters of Orthologous Groups of proteins), Swiss-Prot (a manually annotated and reviewed protein sequence database), KO (KEGG Ortholog database), and GO (Gene Ontology). Unigene expression levels were expressed as fragments per kilobase of transcript per million fragments mapped (FPKM). The FPKM values were calculated using RSE (Li and Dewey, 2011). Differential gene expression analysis of the three groups was performed using the DESeq R package (1.10.1) (Anders and Huber, 2010). The resulting *p*-values were adjusted using Benjamini and Hochberg’s approach to control the false discovery rate. Genes with an adjusted *p*-value <0.05 as determined by DESeq analysis were considered to be differentially expressed.

### Quantitative reverse-transcription PCR assay for validation of gene expression

RT-qPCR primers (Invitrogen, Beijing, China) specific for individual target genes are listed in Supplementary Table 1. RT-qPCR was performed using ABI 7500 Real-Time PCR instrument (Applied Biosystems, Foster City, CA, United States). PrimeScript™ RT reagent kit with gDNA Eraser (TaKaRa, Dalian, China) was used for first-strand cDNA synthesis. Real-time PCR was carried out with the first-strand cDNA template following the manufacturer’s instructions; 18S rRNA gene of *Schizochytrium* sp. FJU-512 served as the internal control.

## Results and discussion

### Determination of optimal malate concentration for biomass, fatty acid accumulation, and DHA production in *Schizochytrium* sp. FJU-512 using shake flask culture

**Figure 1:**
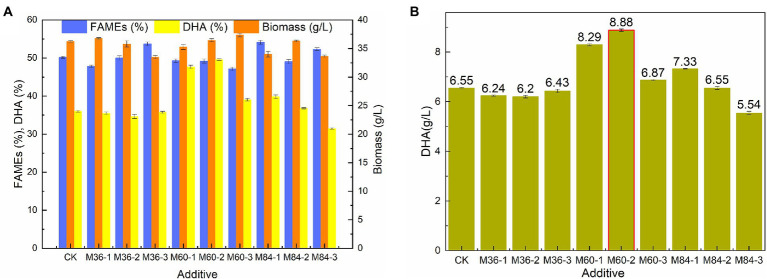
Comparison of biomass, FAME, DHA content, and DHA yield by Schizochytrium sp. FJU-512 shaking cultivated under different malate conditions. Each point is the mean ± SD of three independent experimental replicates. Error bars represent the standard error of means. CK: control group (no malate added); M36, M60, M84: malate added at 36, 60, and 84 h, respectively; −1, −2, −3: 1 g/l, 3 g/l, and 5 g/l of malate, respectively. **(A)** Biomass, FAME (%), and DHA (%) with respect to malate addition and time points. **(B)** DHA (g/L) with respect to malate addition and time points.

Malate has significant effects on the growth and lipid accumulation of *Schizochytrium* sp. (Ren et al., 2009; Zhang et al., 2016). Biomass, lipid, and DHA production by *Schizochytrium* sp. FJU-512 was investigated under three different malate concentrations (1 g/l, 3 g/l, and 5 g/l malate) at 36 h, 60 h, and 84 h, respectively (Figures 1A,B). The findings demonstrated that neither the biomass nor the total fatty acid content were significantly affected by the addition of malate. When malate was added too early (36 h) or too late (84 h), DHA synthesis was not favorable (Figure 1A). DHA content rise from 35.99 to 47.67 and 49.63%, and its titer increased from 6.55 g/l to 8.29 g/l, 8.88 g/l when 1 g/l and 3 g/l malate were added at 60 h, respectively (Figure 1B). Malate, citrate, succinate, and fumarate are significant citric acid cycle intermediates that are essential to microbial. Malate and citrate are advantageous for the fermentation of production since they might speed up cell development. (Ke et al., 2017; Guo et al., 2020; Zhang Y. et al., 2021). In this study, we find malate could affect *Schizochytrium* sp. biomass slightly, but enhance DHA titer significantly. It is essential to understand how *Schizochytrium* sp. synthesizes its fatty acids and produces elevated levels of DHA. Therefore, there is a need to gain insights into oil biosynthesis associate genes that contribute to the improvement of PUFA synthesis.

### Effects of malate on fatty acid accumulation and DHA production in *Schizochytrium* sp. FJU-512 under Fed-batch fermentation

**Figure 2:**
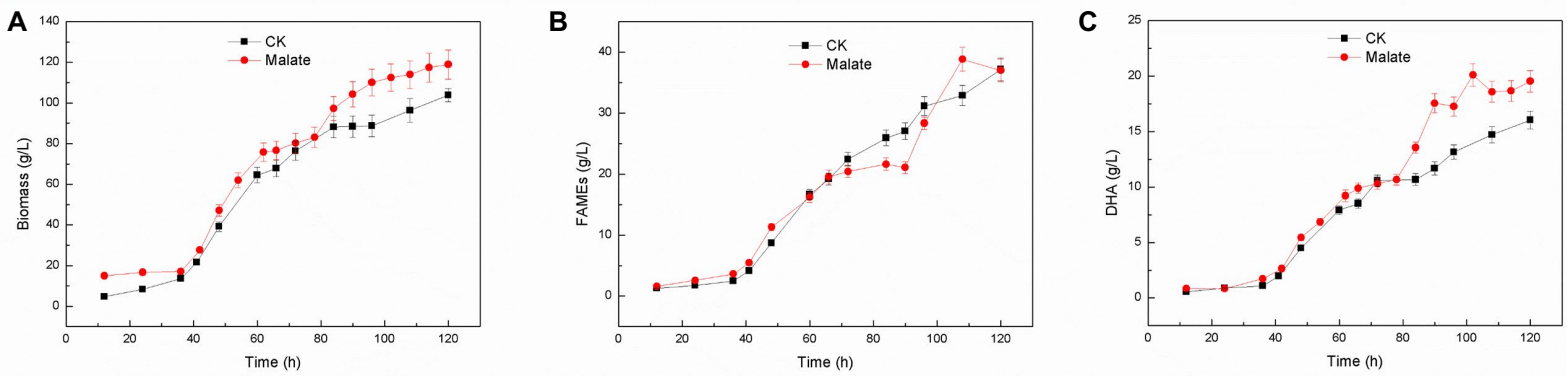
Time course (growth characteristics) of changes in DHA production in *Schizochytrium* sp. FJU-512 grown with or without malate. Each point is the mean ± SD of three independent experiment replicates. Error bars represent the standard error of the means. CK: control group (no malate addition); Malate: 3 g/l malate added at 60 h. **(A)** Growth curve. **(B)** FAME (%) was calculated at different time intervals. **(C)** DHA concentration (g/L) at different time intervals.

Using 3 g/l malate as a supplement for *Schizochytrium* sp. FJU-512 did not show any noticeable effect on cell growth and fatty acid content but significantly affected fatty acid composition, as it increased the proportion of DHA in total fatty acids (Figure 1A). Fed-batch fermentation of *Schizochytrium* sp. FJU-512 was carried out in a 15 l-bioreactor with the addition of 3 g/l malate at 60 h to increase DHA production. The time course analyses of cell growth, lipid accumulation, and DHA production are shown in Figure 2. After 60 h of fermentation, cell growth was shown a slight dip after the supply of malate, but after a short adjustment period, the growth rate appeared slightly higher than that of the control batch without malate addition (Figure 2A). After 120 h, the biomass increased to 118.89 g/l, which was 14.47% higher than that of the control batch (Figure 2A). Similar results were observed with lipid accumulation, where a few fatty acids were degraded in the later stages of fermentation (Figure 2B). Compared to the control batch, the total fatty acid content remained almost unchanged (Figure 2B), but the DHA concentration increased steadily up to 24 h after the addition of malate (from 60 h till 84 h of fermentation) (Figure 2C). The DHA percentage in total fatty acids changed from 43.15 to 47.19% at the end of the fermentation (Figures 2B,C). The final harvested DHA concentration was 19.54 g/l, which was 21.82% higher than that of the control experiment (Figures 2B,C). The results obtained with fed-batch fermentation were consistent with those from the shake flask experiment (Figure 1A).

In general, lipid accumulation in microalgae can be induced by subjecting algal cultures to stress conditions such as nitrogen depletion, changes in light intensity, temperature, salt concentration, etc. (Sun et al., 2018). Temperature is an important factor, it affects both cell growth and fatty acid profile in microorganisms (Hu et al., 2022). Low-temperature conditions triggered the overexpression of *ACC* gene in the DHA accumulator *Aurantiochytrium,* which led to an increase in the accumulation of malonyl-Co A for subsequent fatty acid synthesis (Ma et al., 2015). Transcriptome analysis of glycerol-treated *Schizochytrium* sp. S056 resulted in the upregulation of genes responsible for glycolysis and branched-chain amino acids (BCAAs) metabolism and that of the associated fatty acid synthase (FAS) gene (Chen et al., 2016). The production of DHA may be considerably boosted by the addition of malate during rapid cell growth (Figure 2C), which is probably connected to the regulation of some associated gene expression levels. Further research is needed to reveal the exact mechanism of fatty acid biosynthesis in *Schizochytrium* when malate is used.

### *De novo* assembly and functional annotation of *Schizochytrium* sp. transcriptome

With the use of cDNA libraries created from total RNA of 72 hand 86 h, the gene expression profiles of *Schizochytrium sp*. FJU-512 when malate was introduced at 60 h in fed-batch cultures were examined using the Illumina platform. Samples of *Schizochytrium sp*. FJU-512 grown with and without malate were examined in order to better understand the metabolic pathways that resulted in the accumulation of lipids in the presence of malate. Fatty acid synthesis in *Schizochytrium* sp. is divided into two stages: the lipid rapid accumulation stage, which has the highest lipid accumulation rate, and the lipid turnover stage (Ren et al., 2017). RNA-seq analysis was performed at these two increased points (72 h and 86 h) after malate addition at 60 h. We obtained 200–267 million raw reads from each sample. After cleaning and quality checks, the Illumina sequencing generated 195–261 clean reads. The range of clean base sequencing capacity distribution was 2.92G to 3.92G. The error rate was Q20 (percentage of bases with quality N20 in clean reads), and GC percentages were 0.02–0.03%, 97.56–98.63%, and 51.74–52.60%, respectively. The remaining clean reads were *de novo* assembled into transcripts using Trinity, a short-read sequence-assembling program. After assembly, 62,841 transcripts were generated with an N50 of 1894 nt. Furthermore, 60,949 unigenes for these samples were obtained, with a minimum length of 201 bp, a maximum length of 31,769 bp, and an N50 of 1864 bp. In this study, almost half of the assembled unigenes were short (less than 500 bp, 60.8%), allowing for significantly meaningful matches, but 13,852 (22.7%) unigenes were longer than 1,000 bp.

To annotate the assembled unigenes, all transcriptome sequences were BLAST analyzed against seven different public databases with an E-value threshold of 10^−5^ (1.0E^−5^) for the similarity search. The number of unigenes annotated in at least one database was 32,558 (53.42%), while 4,419 (7.25%) unigenes shared similarities with all seven databases. In addition, 18,594 (30.51%) unigenes were annotated in NCBI Nr; 16,177 (26.54%) in NCBI Nt; 15,745 (25.83%) in KO; 22,351 (36.67%) in SwissProt; 24,588 (40.34%) in Pfam; 9,035 (14.82%) in GO; and 19,480 (31.96%) in COG/KOG. The functionally annotated results in this study were similar to, rather better than, those of other published studies (Chen et al., 2016; Hoang et al., 2016; Ren et al., 2017).

### Changes in the gene transcription profile and functional annotation of DEGs

**Figure 3:**
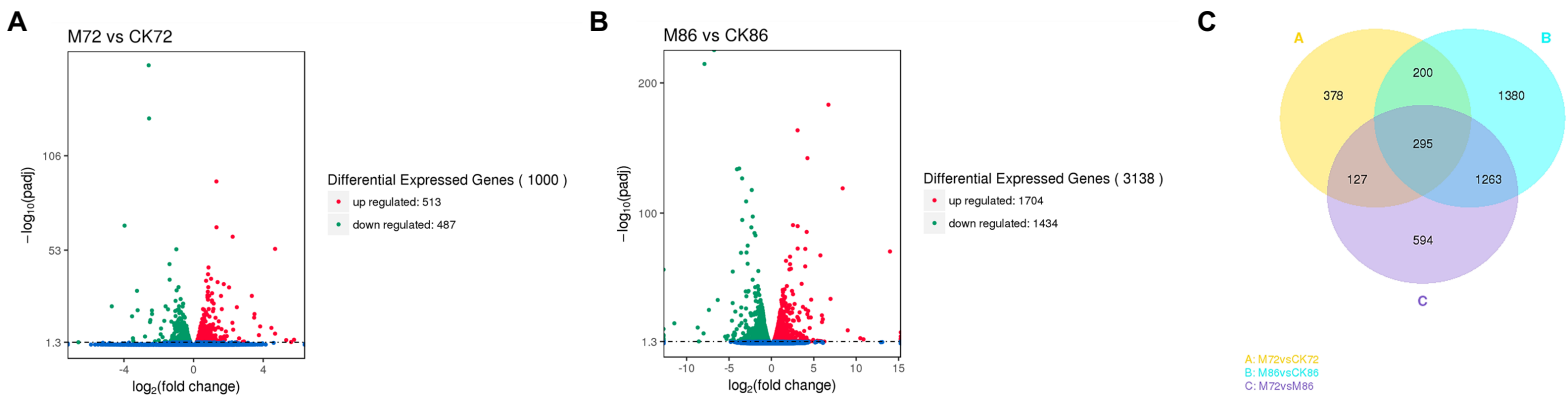
Volcano map and Venn diagram of differentially expressed genes (DEGs). Volcano map of DEGs of **(A)** 72 h samples and **(B)** 86 h samples. The transverse coordinates represent multiple changes in genes expression in different samples, while the longitudinal coordinates represent the significant variations in gene expression; the DEGs are represented as red dots (upregulation) and green dots (downregulation), while blue dots (unchanged). **(C)** Venn diagram showing DEGs among three experimental groups **(A–C)**.

To further investigate the molecular differences between lipid accumulation (72 h) and lipid turnover stages (at 86 h), differences in gene expression were examined at these two stages. After comparing the transcriptional levels of *Schizochytrium* sp. FJU-512 grown under two different conditions, a total of 1,000 unigenes (M72 vs. CK72) and 3,138 unigenes (M86 vs. CK86) with different expression levels were identified by sequence alignment (Figure 3). Among the 1,000 DEGs, 513 were upregulated and 487 were downregulated between M72 and CK72. Among the 3,138 DEGs between M86 and CK86, 1704 were upregulated while 1,434 were downregulated. A Venn diagram was constructed to identify the commonly and exclusively regulated genes during these two stages of fermentation (Figure 3). A comparison of various stages identified 1,000, 3,188, and 2,279 DEGs in the M72 vs. CK72, M86 vs. CK86, and M72 vs. M86 pairs, respectively. The number of DEGs detected in the M72 vs. CK72 pairs was much lower than that in the M86 vs. CK86 pairs, while the number in the M72 vs. M86 pairs was lower than that in the M86 vs. CK86 pairs, indicating a significant difference in the gene expression pattern after malate was added. Understanding this mechanism is particularly important in discerning the biosynthesis of fatty acids in *Schizochytrium* sp. (Bi et al., 2018).

### DEG expression related to NADPH and acetyl-CoA

**Figure 4:**
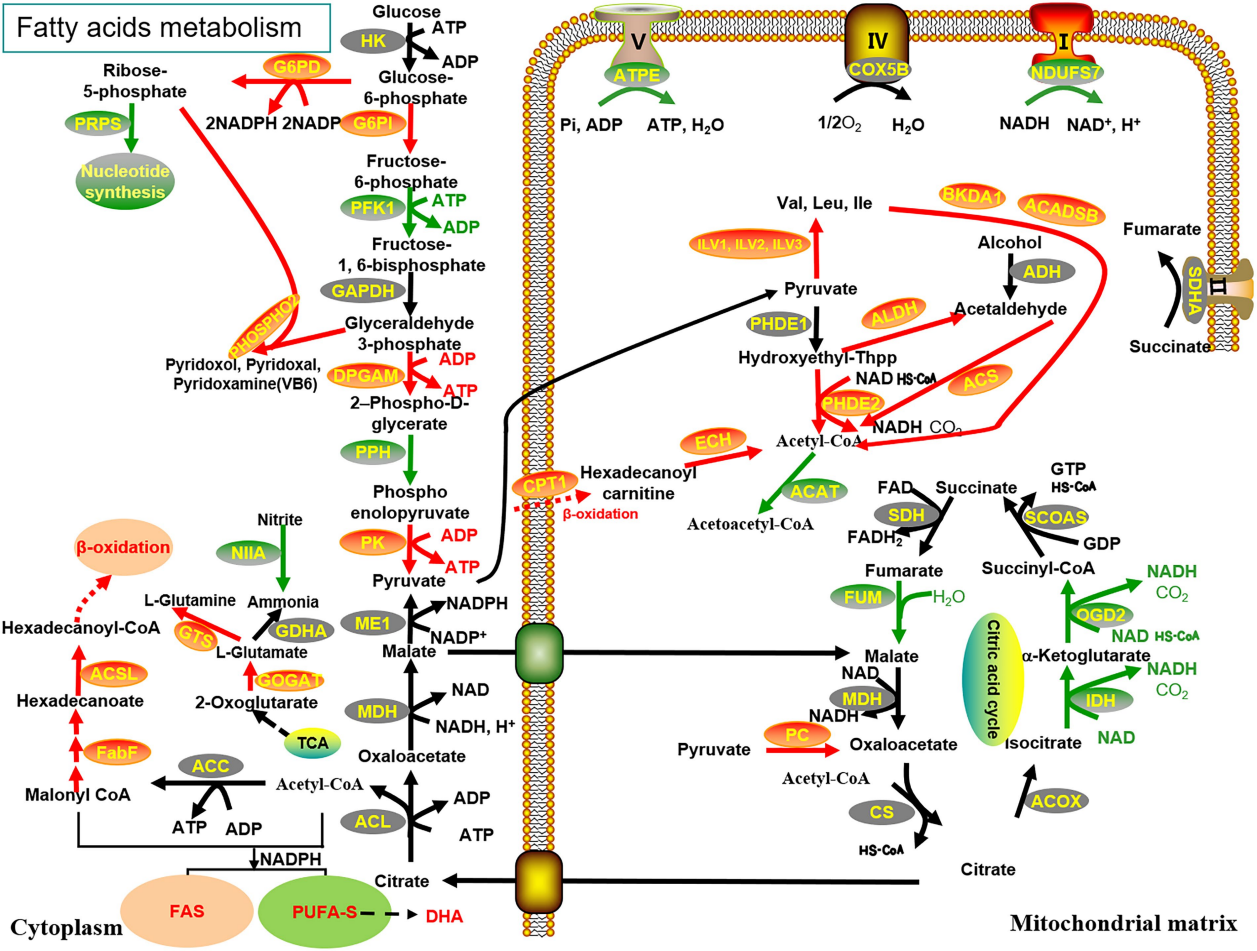
Reconstruction and illustration of the key genes with highly enhanced transcriptional levels and their metabolic processes. The metabolic processes of the TCA cycle, glycolysis pathway, PPP, fatty acid metabolism, β-oxidation, amino acid biosynthesis and degradation, oxidative phosphorylation, nitrogen metabolism, and vitamin B6 synthesis are associated with the fermentation of *Schizochytrium* sp. FJU-512 when malate is added. Red (upregulated), black (no distinct disparities), and green (downregulated); arrows indicate transcriptional regulation of genes: solid line arrows represent a one-step reaction, while dashed ones represent a multi-step reaction. The abbreviations of all genes are detailed in Supplementary Table 2.

The biosynthesis of PUFAs in *Schizochytrium* sp. has been attributed to two pathways, namely, FAS desaturase/elongase and PUFA-S (Metz et al., 2001). However, the activity of Δ^4^ desaturase, which majorly assists in converting DPA (Docosapentaenoic acid) to DHA in the FAS pathway, was not detected in *Schizochytrium* (Huang et al., 2008). Enhancement of lipid accumulation in *Schizochytrium* sp. requires a continuous supply of acetyl-CoA and NADPH (Ren et al., 2009; Ratledge, 2014; Chi et al., 2022). To determine the metabolic pathway through which malate affects DHA production, a comparative transcriptome analysis of *Schizochytrium* sp. FJU-512 was performed under different conditions. The results revealed many changes in gene expression with or without the addition of malate in samples of *Schizochytrium* sp. FJU-512 at 72 h and 86 h, respectively, (Figure 4).

A combination of various treatments and stress conditions can enhance the supply of acyl-CoA and stimulate lipid production in microalgae (Chen et al., 2016; Bi et al., 2018; Liu et al., 2018; Sun et al., 2018). Acetyl-CoA carboxylase (ACC) is a key enzyme in the fatty acid biosynthetic pathway and serves as a fatty acid precursor in both the FAS and PUFA-S pathways (Krivoruchko et al., 2015). However, the transcriptional level of ACC in Schizochytrium sp. FJU-512 remained constant under both conditions. Pyruvate dehydrogenase complex (PDHC) and acetyl-CoA synthetase (ACS) reactions have been correlated with the generation of acetyl-CoA for fatty acid biosynthesis (Lin and Oliver, 2008). Notably, the transcriptional levels of ACS and pyruvate dehydrogenase E2 component (PDHE2) were detected with differences in expression patterns; ACS was downregulated by 1.46-fold at 72 h and upregulated by 1.46-fold at 86 h, while PDHE2 was upregulated by 2.80-fold at 72 h and 3.07-fold at 86 h, further confirming that these key genes are important campaigners for the continuous production of acetyl-CoA. The expression of alcohol dehydrogenase (ADH) was upregulated 3.89-fold at 72 h, and aldehyde dehydrogenase (ALDH) was upregulated 1.14-fold at 86 h. ADH and ALDH are two of the main enzymes that metabolize alcohol. In yeast, the flux flow from ethanol to acetyl-CoA was successfully redirected by the co-expression of the endogenous ADH2, ALDH6, and ACS (Kocharin et al., 2012). Ethanol is an effective inducer to promote astaxanthin accumulation in Schizochytriu. limacinum (Zhu et al., 2022). Therefore, we hypothesized that ADH and ALDH overexpression would further increase the availability of acetyl-CoA.

Acetyl-CoA is produced by various catabolic reactions, including glycolysis, β-oxidation, and degradation of some amino acids (Harris et al., 2005; Zhang Q. et al., 2021). The genes of acyl-CoA oxidase (ACOX) and enoyl-CoA hydratase (ECH), which are involved in the β-oxidation reaction in mitochondria, were found to be downregulated 1.15-fold at the rapid accumulation stage of fatty acids (72 h, CK vs. malate) and upregulated 1.20-fold at the turnover stage of fatty acids (86 h, CK vs. malate). The expression levels of key enzymes that aid the transport of fatty acids into the mitochondria for β-oxidation demonstrated the downregulation of carnitine *O*-acyltransferase 2 (C PT2) by 1.27-fold at 72 h and the upregulation of carnitine *O*-acyltransferase 1 (CPT1) by 1.24-fold at 86 h. This may be an indication that partially saturated fatty acids were transformed into PUFAs such as DPA and DHA.

Nitrogen deprivation can increase the generation of PUFAs by rerouting the glycolytic flow of carbon backbones into fatty acid biosynthesis because certain amino acids, such as BCAAs, when broken down, release acetyl-CoA (Chang et al., 2021). The transcriptome data in this study showed that the pathways for Val-Leu-Ile metabolism were upregulated when malate was added, which is another pathway for acetyl-CoA generation. The expression of enzymes at 72 h and 86 h included acetolactate synthase I/II/III large subunit (ILVB, ILVG, and ILVI), which were upregulated by 3.28 and 8.18-fold; 2-oxoisovalerate dehydrogenase E1 component alpha subunit (BKDA1), which was upregulated by 1.02-and 1.23-fold; and short/branched-chain acyl-CoA dehydrogenase (ACADSB), which were upregulated 1.18-and 1.46-fold. Generally, malate enhanced both synthesis and the degradation of branched-chain amino acids, forcing carbon flux to acetyl-CoA for DHA production and TCA intermediate compounds. These results are similar to those of a previous study under low temperatures to culture *Schizochytrium* (Hu et al., 2020).

Malate has an important role as the intermediate in fatty acid biosynthesis due to its pool regulates the activity of malic enzyme and the transportation of citrate in mitochondria (Wang et al., 2021). Malic enzyme (ME) is a key enzyme regulating the accumulation of lipids, catalyzing the conversion of l-malate to pyruvate with concomitant NADPH production, thereby supplying reducing power for fatty acid biosynthesis (Liang and Jiang, 2015). However, the current study showed that *me* gene was not significantly upregulated when malic acid was added at 72 h and 86 h. This is likely due to the high ME expression levels, which are the same whether malate is induced or not. It’s also possible that an alternative route of NADPH production existed. An obvious alternative is enzymes associated with the pentose phosphate pathway (PPP): glucose-6-phosphate dehydrogenase (G6PD) and 6-phosphogluconate dehydrogenase (6GPD) (Ratledge, 2014). The transcriptome data in this study showed that PPP metabolism, which generates NADPH, is promoted when malate is used. From 72 h to 86 h of fermentation, *g6pd* changed from downregulation by 1.22-fold to upregulation by 3.08-fold, which proved its vital role in NADPH supply at the fatty acid turnover stage (which begins at 86 h). Bi et al. attributed the upregulation of *Schizochytrium* sp. HX-308 ATP citrate lyase (ACL) to the accumulation of NADPH (Bi et al., 2018). However, there was no change in *acl* expression in our study, suggesting that upregulation of *g6pd* was the main contributor to the supply of additional NADPH.

### DEGs related to fatty acid synthesis

*Schizochytrium* can store substantial amounts of PUFAs under conducive fermentation conditions, making it suitable for sustainable DHA production; the expression of PUFA synthases (Pufa A/B/C) increases under cold treatment (Ma et al., 2015). In the transcriptome study, there was no slightly different expression of the PUFA-S genes (*pks*A, *pks*B, *pks*C), which are directly involved in the biosynthesis of DHA. This result is consistent with the transcriptome analysis of glycerol-fed *Schizochytrium* (Chen et al., 2016). We speculate that the attribution of DHA production in *Schizochytrium* to the intense activity of DHA-directed synthetase, whose high expression levels indicated that the associated genes do not need to be modified to promote DHA yield. By contrast, the expression of certain genes related to other pathways was more significant in increasing DHA production. Such candidate genes encoding enzymes related to DHA biosynthesis were mined from the transcriptome data.

Long-chain acyl-CoA synthetase (ACSL) activates long-chain and very-long-chain free fatty acids to form acyl-CoAs; increasing the size of the acyl-CoA pool by enhancing ACSL activity would improve the production and also help modify the composition of fatty acid-derived compounds such as triacylglycerol (Mashek et al., 2007; Xu et al., 2019). Besides, some ACSLs are also fatty acid transport proteins (FATPs) owing to their second function as mediators of transmembrane movement of fatty acids in yeast and mammalian systems (Pulsifer et al., 2012). According to the KEGG annotation of the *Schizochytrium* sp. transcriptome in the present study, *acsl* genes were predicted to be involved in fatty acid metabolism, upregulated by 1.04-and 1.21-fold (at 72 h and 86 h). Additionally, the expression of 3-oxoacyl-[acyl-carrier-protein] synthase 2 (FabF) increased by 1.29-and 1.83-fold at 72 h and 86 h, respectively. DHA and DPA are known to be synthesized by PUFA-S, whereas palmitic acid (C16:0) is produced by fatty acid synthase (FAS) (Metz et al., 2001). FabF is a key element in type II fatty acid synthesis, which is catalyzed by a “ping-pong” mechanism, and these enzymes have substrate-binding sites for acetyl-CoA and malonyl-ACP (Abbadi et al., 2000). The overexpression of *fabF* increased intracellular malonyl-CoA (Masuo et al., 2022). Therefore, malate addition resulted in the upregulation of *fabF*, which probably leading to PUFA-S exploit the improvement of malonyl-CoA and enhancement DHA production in *Schizochytrium* sp. FJU-512.

### Analysis of the cellular processes associated with the addition of malate

Transcriptomic and metabolomic analysis revealed some pathways including carbohydrate metabolic pathways, nitrogen metabolism, fatty acid β-oxidation, and glycerophospholipid metabolism pathways, are related to lipid biosynthesis (Chen et al., 2016; Ren et al., 2017; Liu et al., 2018). On the basis of transcriptome data, the metabolisms of the TCA cycle, biotin metabolism, glycolysis/gluconeogenesis, fatty acid degradation, oxidative phosphorylation, pyruvate metabolism, nitrogen metabolism, vitamin B6 metabolism, and PPP were investigated. The 6-phosphofructokinase 1 (pfk1) gene, which is involved in glycolysis, was downregulated 1.49 and 1.26-fold at 72 and 86 h, respectively, when *Schizochytrium* sp. FJU-512 was cultivated with malate. Since the effect of citrate on glycolysis is the direct inhibition of phosphofructokinase, the inhibition of glycolysis by fatty acids can be attributed to low phosphofructokinase activity (Yoshino and Murakami, 1982). Pyruvate kinase (PK) catalyzes the transfer of a phosphate group from phosphoenolpyruvate (PEP) to adenosine diphosphate (ADP), generating one molecule of pyruvate and one molecule of ATP (Gupta and Bamezai, 2010). The *pk* gene is downregulated 1.51-fold at 72 h and upregulated 2.01-fold at 86 h after the addition of malate. Thus, inhibiting glycolysis pathways causes carbon metabolism to be diverted toward PPP, which produces more NADPH; in contrast, increasing pyruvate during the lipid conversion stage is advantageous for the buildup of fatty acids.

Intermediates of the TCA cycle can be precursors as well as cofactors for fatty acid synthesis (Muhlroth et al., 2013). Transcriptome analysis of the TCA cycle revealed that most of the gene expression was unchanged or even partly downregulated. The activities of isocitrate dehydrogenase (IDH), 2-oxoglutarate dehydrogenase E2 component (OGD2), and fumarase (FUMC) were slightly decreased during the fatty acid turnover stages. These results demonstrate that *Schizochytrium* sp. FJU-512 generates DHA at higher levels than typical without much assistance from the TCA cycle. In addition, the results showed that the reaction catalyzed by glutamate synthase (GOGAT) and glutamine synthetase (GTS), which were involved in glutamate metabolism, were upregulated by 1.07-and 2.16-fold at 72 h and 86 h, respectively, which may have contributed to protection during unfavorable cultivation environments of *Schizochytrium* sp. FJU-512 during the late fermentation stage, according to our previous study (Qi et al., 2017). Transcriptomic studies showed that some genes related to nitrogen metabolism, such as glutamate dehydrogenase (NADP^+^) (GDHA) and nitrite reductase (NAD(P)H) (NIIA), were upregulated 1.29-and 1.94-fold at 72 h and 86 h, respectively, and downregulated 1.07-and 1.66-fold, suggesting that cell growth was accompanied by rapid accumulation of lipids. Simultaneously, more precursors and energy were used to synthesize PUFAs during the lipid turnover stage. It was also observed that genes associated with oxidative phosphorylation, such as NADH dehydrogenase (ubiquinone) Fe-S protein 7 (NDUFS7), succinate dehydrogenase (ubiquinone) flavor protein subunit (SDHA), F-type H^+^-transporting ATPase subunit epsilon (ATPE), cytochrome C oxidase subunit 5b (COX5B), were all upregulated at 72 h and downregulated at 86 h. These observations indicate that a large quantity of energy was generated during the period of rapid accumulation of fatty acids in preparation for DHA transformation. In the case of *Oncorhynchus mykiss* supplemented with vitamin B6, there was a significant increase in the percentage of long-chain PUFAs, particularly DHA (Maranesi et al., 2005). Transcriptomic studies of malate used in *Schizochytrium* sp. FJU-512 showed 1.02-and 1.22-fold upregulation of the pyridoxal phosphate phosphatase (PHOSPHO2) gene, which is responsible for the synthesis of vitamin B6. As one of the cofactors of the enzyme synthetase in the process of lipid biosynthesis, vitamin B6 is crucial for the production of unsaturated fatty acids.

### RT-qPCR validation of transcriptome analysis

**Figure 5:**
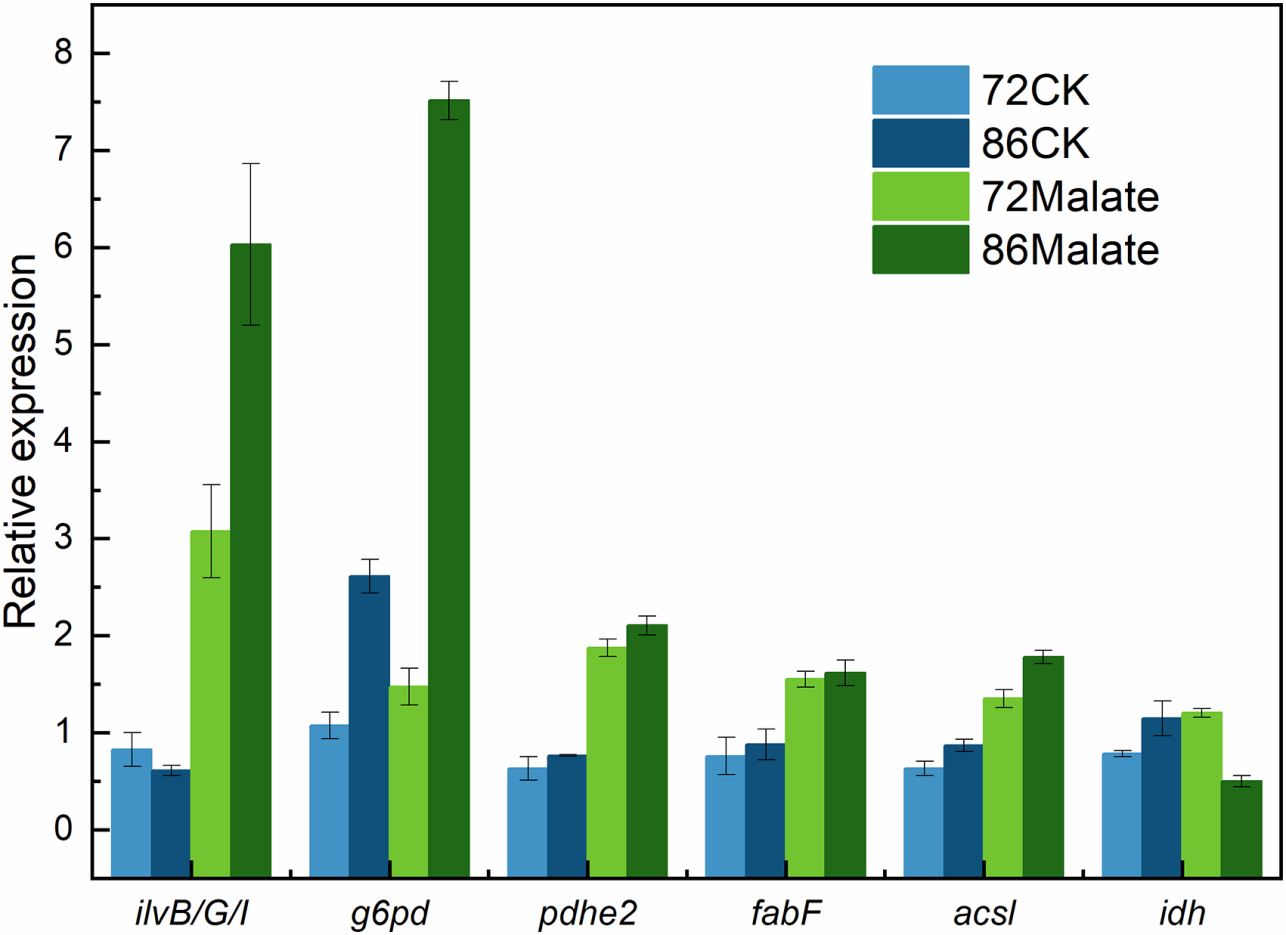
Validation of the gene expression profiles of the *ilv*B*, ilv*G, *ilv*I*, g6pd, pdhe2, fab*F*, acsl,* and *idh* genes by quantitative reverse-transcription PCR. Light blue represents the control group at 72 h, which is the expression level of 18 s rDNA; At 86 h, Dark blue represents the control group. While dark green indicates each gene’s expression at 86 h, light green indicates each gene’s expression at 72 h.

Six genes (*ilv*B*/G/*I*, g6pd, pdhe2, fab*F*, acsl,* and *idh*) associated with fatty acid biosynthesis that are differentially expressed between malate and control groups at 72 h and 86 h were analyzed to confirm their expression profiles to validate the transcriptome analysis data (Figure 5). The gene expression levels detected by RT-qPCR were consistent with those obtained by transcriptome analysis, thus, confirming that the data from the *de novo* transcriptome sequencing platform were reliable.

### Effect of additives on the growth and the fatty acid profile of *Schizochytrium* sp. FJU-512

**Figure 6:**
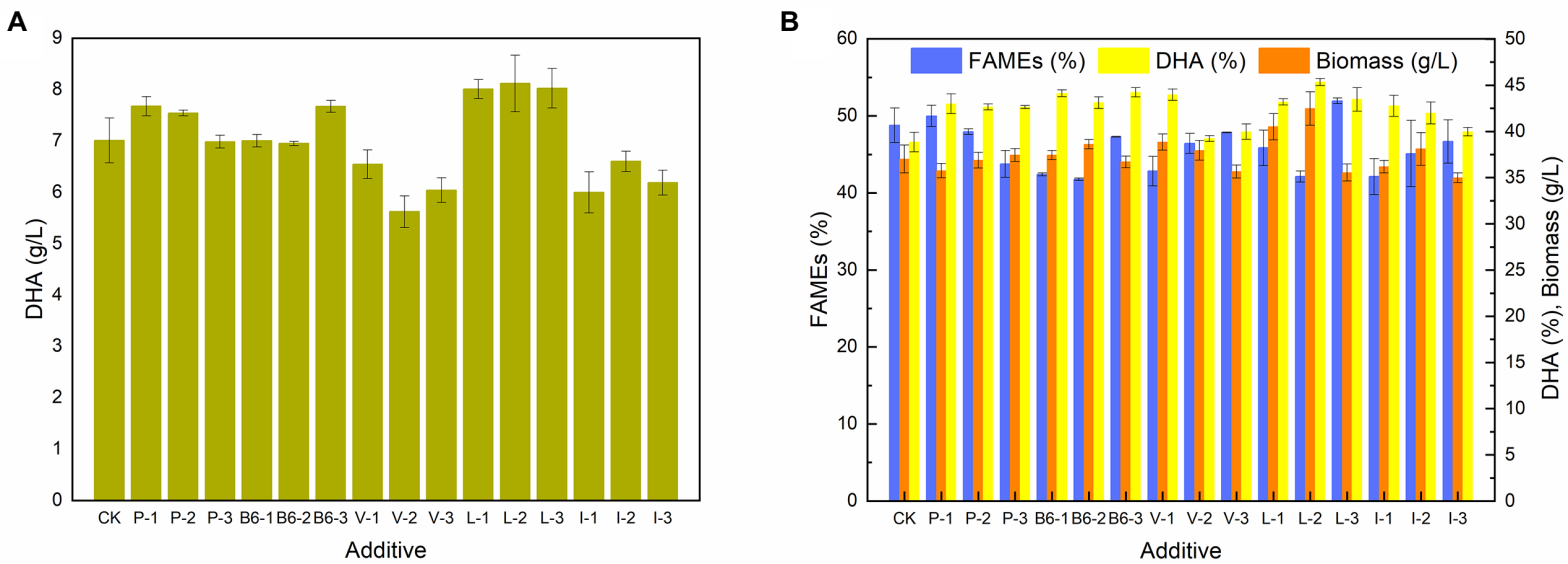
Effect of additives on growth, FAME, DHA content, and DHA yield of *Schizochytrium* sp. FJU-512 when shake culture at 72 h. Each point is the mean ± SD of three independent experimental replicates. Error bars represent standard errors of the means. CK: control group (no additive added); P-1, P-2, P-3: addition of 1, 2.5 and 4 mmol/l sodium pyruvate, respectively; B6-1, B6-2, B6-3: 0.05, 0.25, and 0.45 mg/ml of Vitamin B6, respectively; V-1, V-2, V-3: 0.06, 0.30, and 0.54 mg/ml of Val, respectively; L-1, L-2, L-3: 0.06, 0.30, and 0.54 mg/ml of Leu, respectively; I-1, I-2, I-3: 0.06, 0.30, and 0.54 mg/ml of Ile, respectively. **(A)** Concentration of DHA (g/L). **(B)** Concentration of biomass, FAME (%), and DHA (%).

To further confirm the results of the transcriptome analyses, certain major metabolic products, including sodium pyruvate, vitamin B6, Val, Leu, and Ile, were added to the culture of *Schizochytrium* sp. FJU-512 at 72 h in a 250 ml conical flask. Figure 6 shows the effects of additives on the amount of biomass, lipids, and DHA content in *Schizochytrium* sp. FJU-512. The DHA concentration of *Schizochytrium* sp. FJU-512 increased under specific concentrations of additives. DHA yield increased from 7.01 g/l to 7.68 g/l, 7.67 g/l, 7.31 g/l, 8.11 g/l, and 7.21 g/l with the addition of 1 mmol/l sodium pyruvate, 0.45 mg/ml Vitamin B6, 0.06 mg/ml Val, 0.54 mg/ml Leu and 0.54 mg/ml Ile, respectively. Meanwhile, as shown in Figure 6B, DHA content significantly increased under all operating conditions. In addition, the total fatty acid content showed different degrees of reduction, excluding that of 1 mmol/l sodium pyruvate and 0.6 mg/ml Leu. These results suggest the importance of pyruvate, vitamin B6, Val, Leu, and Ile metabolic pathways in regulating the lipid turnover phase of *Schizochytrium* sp. FJU-512. These data further validated the transcriptome analysis results.

## Conclusion

In this study, we found that malate can significantly promote DHA accumulation in *Schizochytrium* sp. FJU-512, where the titer increased from 16.01 g/l to 19.54 g/l when 3 g/l malate was supplied at 60 h in a fed-batch fermentation set-up. Our transcriptomic analysis results showed that pyruvate dehydrogenase E2 component (PDHE2) and acetolactate synthase I/II/III large subunit (ILVB, ILVG, and ILVI) were the major providers of acetyl-CoA, while glucose-6-phosphate dehydrogenase (G6PD) was the chief contributor of NADPH. The results indicate the presence of some interesting pathways in *Schizochytrium* sp. FJU-512 is responsible for enhanced DHA yield by upregulation of the acetyl-CoA biosynthesis system.

## Data availability statement

The original contributions presented in the study are included in the article/Supplementary material, further inquiries can be directed to the corresponding authors.

## Author contributions

MZ: Methodology, validation, investigation, resources, writing—original draft. YG: Methodology, validation, investigation. CY: Methodology, investigation. JW: Methodology, investigation. KW: Methodology, validation, investigation. QL: Methodology, investigation. YH: Methodology, software. ZG: Writing—review and editing. HZ: Resources, writing—review and editing, supervision. JH: Methodology, writing—review and editing, supervision. LL: Resources, supervision, funding acquisition. All authors contributed to the article and approved the submitted version.

## Funding

This work was supported by the National Key R&D Program of China under grant no. 2021YFA0910501, the National Natural Science Foundation of China under grant nos. 32101898, 31870039, and 32170069, and the Natural Science Foundation of Fujian Province under grant no. 2022J01635.

## Conflict of interest

The authors declare that the research was conducted in the absence of any commercial or financial relationships that could be construed as a potential conflict of interest.

## Publisher’s note

All claims expressed in this article are solely those of the authors and do not necessarily represent those of their affiliated organizations, or those of the publisher, the editors and the reviewers. Any product that may be evaluated in this article, or claim that may be made by its manufacturer, is not guaranteed or endorsed by the publisher.
