# Supplementary material for: Transcriptome analysis of malate-induced Schizochytrium sp. FJU-512 reveals a novel pathway for biosynthesis of docosahexaenoic acid with enhanced expression of genes responsible for acetyl-CoA and NADPH accumulation
Source: Front Microbiol. 2022 Oct 10;13:1006138. doi: 10.3389/fmicb.2022.1006138 (PMC9589357; doi:10.3389/fmicb.2022.1006138)
Supplement: Supplementary file 1 [file Data_Sheet_1.docx]

Supplementary Table 1. Primers used in this study

| primers | sequence (5′ to 3′) | amplicon length (bp) |
| --- | --- | --- |
| *ilv* B/G/I-f | GAGGGCTGGATGTCCTACTG | 284 |
| *ilv* B/G/I-r | TCGTTTGGTCAGTCGCAGT |  |
| *g6pd*-f | AGATCAACGCTATTTGGGACAT | 199 |
| *g6pd*-r | GTTTCAAGCCCAAGTCTCTCTT |  |
| *pdhe2*-f | GTTGTCCTGGGCATCCTCT | 186 |
| *pdhe2*-r | GAGATTGAGACCGACAAGGC |  |
| *fab*F-f | TTCTCGCTCGCGGGATAT | 288 |
| *fab*F-r | GGACGAACTTTCTGACACGGT |  |
| *acsl*-f | GCGACCACTGTCTTCAATCTC | 230 |
| *acsl*-r | GAGCCGACAGCGTTGAGTAT |  |
| *idh*-f | TGGACTGACGGAGGCTTTG | 225 |
| *idh*-r | TCCGAGCGGGTTGAGACT |  |
| 18S-f | CACCCGAGTTCTGCCTCT | 268 |
| 18S-r | CGTCCCTGCCCTTGTAC |  |

Supplementary Table 2. Abbreviations of enzymes from comparative transcriptome analyses

| Abb | Enzymes | Abb | Enzymes |
| --- | --- | --- | --- |
| HK | hexokinase | NDUFS7 | NADH dehydrogenase (ubiquinone) Fe-S protein 7 |
| G6PI | glucose-6-phosphate isomerase | SDHA | succinate dehydrogenase (ubiquinone) flavor protein subunit |
| PFK1 | 6-phosphofructokinase 1 | ATPE | F-type H+-transporting ATPase subunit epsilon |
| GAPDH | glyceraldehyde-3-phosphate dehydrogenase | COX5B | cytochrome c oxidase subunit 5b |
| DPGAM | 2,3-bisphosphoglycerate-dependent | ACOX | acyl-CoA oxidase |
| PPH | phosphopyruvate hydratase | IDH | Isocitrate dehydrogenase |
| PK | pyruvate kinase | OGDC | 2-oxoglutarate dehydrogenase E1 component and 2-oxoglutarate dehydrogenase E2component |
| G6PD | glucose-6-phosphate dehydrogenase | SCOAS | succinyl-CoA synthetase |
| PRPS | ribose-phosphate pyrophosphokinase | SDH | Succinate dehydrogenase |
| PHOSPHO2 | pyridoxal phosphate phosphatase PHOSPHO2 | FUM | fumarase |
| ACL | ATP citrate lyase | CS | citrate synthetase |
| MDH | Malate dehydrogenase | PC | pyruvate carboxylase |
| ME1 | malic enzyme 1 | ACAT | acetyl-CoA acetyltransferase |
| ACC | Acetyl-CoA carboxylase | PDHE1 | pyruvate dehydrogenase E1 component |
| FabF | 3-oxoacyl-[acyl-carrier-protein] synthase 2 | PDHE2 | pyruvate dehydrogenase E2 component |
| ACSL | long-chain acyl-CoA synthetase | ALDH | aldehyde dehydrogenase |
| GOGAT | glutamate synthase (NADH) | ACS | acetyl-CoA synthetase |
| GTS | glutamine synthetase | ILVB/G/I | acetolactate synthase I/II/III large subunit |
| GDHA | glutamate dehydrogenase (NADP+) | BKDA1 | 2-oxoisovalerate dehydrogenase E1 component alpha subunit |
| NIIA | nitrite reductase (NAD(P)H) | ACADSB | short/branched-chain acyl-CoA dehydrogenase |
| CPT1/2 | carnitine O-palmitoyltransferase 1/2 | ADH | alcohol dehydrogenase |
| ECH | enoyl-CoA hydratase |  |  |
